# The Differential Modulatory Effects of Potassium Supplementation on Blood Pressure, Vascular Reactivity, Glomerular Filtration Rates, and Oxidative Stress in Different Experimental Hypertensive Models

**DOI:** 10.3390/nu17111865

**Published:** 2025-05-29

**Authors:** Chukwuemeka R. Nwokocha, Javier Palacios, Melissa Kaydeen Reid, Nikolai Javier Nunes, Wesley Gray, Donovan McGrowder, Nelson N. Orie, Momoh A. Yakubu

**Affiliations:** 1 Department of Basic Medical Sciences (Physiology Section), The University of the West Indies, Mona, Kingston 7, Jamaica; melkreid@gmail.com (M.K.R.); nikolaijnunes@gmail.com (N.J.N.); 2 Department of Environmental Toxicology, Southern University, Baton Rouge, LA 70807, USA; wesley_gray@subr.edu; 3 Química y Farmacia, Facultad de Ciencias de la Salud, Universidad Arturo Prat, Iquique 1110939, Chile; 4 Department of Pathology, The University of the West Indies, Mona, Kingston 7, Jamaica; dmcgrowd@yahoo.com; 5 Department of Inflammation and Rare Disease, Division of Medicine, University College London, London NW3 2PF, UK; n.orie@ucl.ac.uk; 6 Department of Environmental & Interdisciplinary Sciences, Texas Southern University, Houston, TX 77004, USA

**Keywords:** potassium supplementation, L-NAME model of hypertension, DOCA-salt model of hypertension, vascular reactivity, electrolyte balance, nutrition

## Abstract

High-sodium/low-potassium in the modern diet, potassium excretion, and sodium retention have all been implicated in hypertension. **Objectives**: This study investigated the differential effects of potassium (K⁺) supplementation on blood pressure, renal function, and oxidative stress in two experimental hypertensive rat models: L-NAME-induced (nitric oxide synthase inhibitor-induced hypertension presenting with reduced NO bioavailability, endothelial dysfunction, vasoconstriction) and DOCA-salt-induced hypertension (deoxycorticosterone acetate + salt mimics volume-dependent hypertension of hypermineralocorticoidism, low renin, high sodium retention and severe cardiac fibrosis and oxidative stress). **Methods**: Male Sprague Dawley rats were treated with L-NAME or DOCA-salt, with or without 0.75% KCl dietary supplementation for eight weeks. Blood pressure, vascular reactivity, serum electrolytes, renal function markers, and malondialdehyde (MDA) levels were evaluated. **Results**: Potassium supplementation significantly reduced (20%) mean arterial pressure and (80%) oxidative stress markers in the L-NAME model but not in the DOCA-salt model. In both hypertensive models, K⁺ reduced (15%) vascular contractile response to phenylephrine, though it did not improve acetylcholine-induced vasodilation. Notably, K⁺ supplementation improved glomerular filtration rate (eGFR), sodium–potassium ratio, and renal biomarkers (urea and creatinine) in the L-NAME model, suggesting nephroprotection. However, in the DOCA-salt group, these markers either remained unchanged or worsened. **Conclusions**: These findings indicate that the antihypertensive and renoprotective effects of potassium are model-specific and depend on the underlying pathophysiological mechanisms, such as nitric oxide bioavailability and mineralocorticoid sensitivity. Dietary potassium may be more effective in patients with endothelial dysfunction-dominant hypertensive subtypes compared with volume-dependent hypertension and may call for K⁺ supplementation studies to be stratified by hypertension subtype.

## 1. Introduction

High-sodium/low-potassium in the modern diet, potassium excretion, and sodium retention have all been implicated in hypertension [1]. These factors are linked with changes in salt sensitivity and differential potassium handling by the kidneys and may contribute to the racial differences seen with hypertension presentation [2]. On the other hand, potassium supplementation has the potential to reverse the salt balance and restore normal blood pressure [3], although the mechanism is not well understood [4]. This beneficial effect of potassium supplementation could be related to decreased stimulation of the renin–angiotensin–aldosterone system, leading to decreased vascular resistance and blood pressure [5,6]. Potassium supplementation can also modulate vasodilatation and baroreceptor sensitivity [7] to impact blood pressure.

L-NAME and DOCA-salt uninephrectomy (DOCA-UNX) models are widely used to study hypertension in experimental animals, particularly in rats. However, they differ significantly in their mechanisms, induction protocols, and physiological outcomes. L-NAME stands for NG-nitro-L-arginine methyl ester, a nitric oxide synthase (NOS) inhibitor. L-NAME hypertension is characterized by increased renin–angiotensin activity [8] and salt sensitivity [6,9,10,11], inhibiting nitric oxide (NO) production and leading to vasoconstriction, increased peripheral resistance, cardiac hypertrophy, renal damage, and oxidative stress [12,13]. Nitric oxide production inhibition will lead to afferent arteriole vasoconstriction and reduced glomerular capillary pressure and glomerular filtration rates (GFRs) through reduction in renal blood flow (RBF). Chronic L-NAME treatment leads to glomerular ischemia, mesangial expansion, and glomerulosclerosis [14]. The DOCA-UNX model involves deoxycorticosterone acetate (DOCA) administration (a synthetic mineralocorticoid), usually combined with salt loading and unilateral nephrectomy (UNX). Hypertension arises due to volume expansion [10,11,15], mineralocorticoid excess, sodium retention, vascular remodeling, and suppressed renin–angiotensin system (RAS) activity [16]. As such, this model responds to aldosterone antagonists (e.g., spironolactone), diuretics, and agents that reduce fibrosis or inflammation [17]. Unilateral nephrectomy causes a loss of half of nephron mass, while DOCA + salt leads to volume expansion, hypertension, and glomerular hyperfiltration in the remaining nephrons. Chronic hypertension is due to glomerular capillary hypertension, podocyte injury, and eventually focal segmental glomerulosclerosis (FSGS) [10,11,18]. 

Studies on the molecular mechanisms by which K+ decreases blood pressure were recently reviewed [5]. In spontaneously hypertensive rats, potassium supplementation is reported to decrease vascular tone through decreased production of vasoconstrictors and increased smooth muscle sensitivity to nitric oxide [19]. Salt-sensitive hypertension is observed in African Americans and the elderly and parallels the DOCA-salt model, thus providing a strong justification for K⁺-rich diets in our experimental hypertensive rat model study. Endothelial dysfunction is observed in smokers and diabetics and parallels the L-NAME model [20]. The aim of this study was to use the two different models (L-NAME and DOCA-salt) of hypertension to gain further insight into the mechanism of the blood pressure-lowering effect of potassium supplementation.

## 2. Materials and Methods

### 2.1. Animals

Male adult Sprague Dawley rats (30), weighing 180–300 g (12 weeks old), were randomly divided into six groups of 5 animals each. The first group consisted of rats treated with vehicle (control), the second group of rats was treated with potassium supplementation (0.75% *w*/*w* KCl in the diet) for 8 weeks, the third group was treated with 40 mg/kg b.w L-NAME, 40 mg/kg b.w L-NAME plus (0.75% *w*/*w*) potassium supplementation in the diet was given to the fourth group, the fifth group was uninephrectomized and implanted with a 21-day slow-release 200 mg DOCA pellet and given 0.9% *w/v* NaCl water ad libitum, and the sixth group was uninephrectomized and implanted with a 21-day slow-release 200 mg DOCA pellet, given 0.9% *w/v* NaCl plus (0.75% *w*/*w*) potassium supplementation in the diet. All animals were acquired from the University of the West Indies animal house and received standard rat chow for the duration of the experiment. All animal care and handling procedures were performed in accordance with the Animal Scientific Procedures Act of 1986, following ethical approval from the UWI/FMS Ethics Committee (AN 16, 13/14) on the 5 February 2014.

### 2.2. Induction of Hypertension and Blood Pressure Recording

L-NAME and DOCA-salt were used to induce hypertension. L-NAME hypertension was induced by gavage feeding of 40 mg/kg daily for 8 weeks. DOCA-salt hypertension was induced through uninephrectomy under anesthesia, by removing the left kidney through a lateral abdominal incision, ligation of the left renal vessels, and implanting a DOCA pellet (200 mg) subcutaneously. The incision was then sutured with sterile suture needles. These rats received 0.9% NaCl water ad libitum [21]. The rats were randomized into two groups: DOCA-salt hypertensive control and DOCA-salt hypertensive rats with potassium supplementation (0.75% *w*/*w* KCl diet) for 8 weeks. Diastolic and systolic blood pressure measurements were taken using the tail-cuff method at the same time every day (CODA, Kent Scientific, Torrington, CT, USA) as described earlier in our laboratory [22].

### 2.3. Isolation of Aortic Rings—Vascular Reactivity Experiments

At the end of the supplementation period, animals were sacrificed via decapitation. The aorta was removed and cleaned of connective tissues and then cut into rings (2 mm) and mounted in 20 mL organ baths at 37 °C containing a modified Kreb’s physiological salt solution of the following composition (×10^−3^ M): NaCl 112; KCl 5; CaCl_2_ 1.8; MgCl_2_ 1, NaHCO_3_ 25; KH_2_PO_4_ 0.5; NaH_2_PO_4_ 0.5; glucose 10; pH 7.4 gassed with 95% O_2_ and 5% CO_2_. The aortic rings were connected to isometric force transducers, which were coupled to a computerized data acquisition system, AcqKnowledge 3.9.1.6 computer program (Biopac Systems Inc., Goleta, CA, USA), for tension recordings. After a 60 min equilibration period, the aortic rings were stabilized through three successive near-maximal contractions with KCl (6 × 10^−2^ M) for 10 min, in accordance with previous studies [21].

Cumulative concentration–response curves were constructed for a vasoconstrictor, phenylephrine (PE), and a vasodilator, acetylcholine (ACh), to determine vascular reactivity.

### 2.4. Serum Electrolyte, Urea, and Creatinine Analyses

Serum was collected after centrifugation of blood at 6000 rpm for 10 min and stored (−80 °C) for measurement of urea, creatinine, and electrolytes. The potentiometric detection technology (ARCHITECT^®^ c8000 System, Abbott Architect, Abbot Park, IL, USA) method was used in the analysis of the serum at the Chemical Pathology Laboratory, Department of Pathology, University of the West Indies.

### 2.5. Serum Malondialdehyde Level Assay

The serum level of malondialdehyde (MDA), which is a product of lipid peroxidation, was determined using the method of Ohkawa [23]. Briefly, serum samples (400 mL) with 200 mL of mixture solution added (8.1% sodium dodecyl sulphate, 1.5 mL of acetic acid 20% pH 3.0, 1.5 mL of thiobarbituric acid 0.8%, 300 mL of butylated hydroxytoluene 4%, and 300 mL of water) were heated at 95 °C for 1 h. The absorbance was measured at 532 nm.

### 2.6. Statistical Analysis

Statistical analysis was performed with the GraphPad Prism 5 software program. Values and data were expressed as the mean ± standard error of the mean. One- or two-way analysis of variance (ANOVA) was carried out to detect significant differences, followed by the Bonferroni test. A *p*-value of <0.05 was considered statistically significant. The sensitivity of the agonists is expressed as pEC_50_ (−log EC_50_).

## 3. Results

### 3.1. Potassium Supplementation Differentially Lowered Blood Pressure in Hypertensive Rats

**Figure 1:**
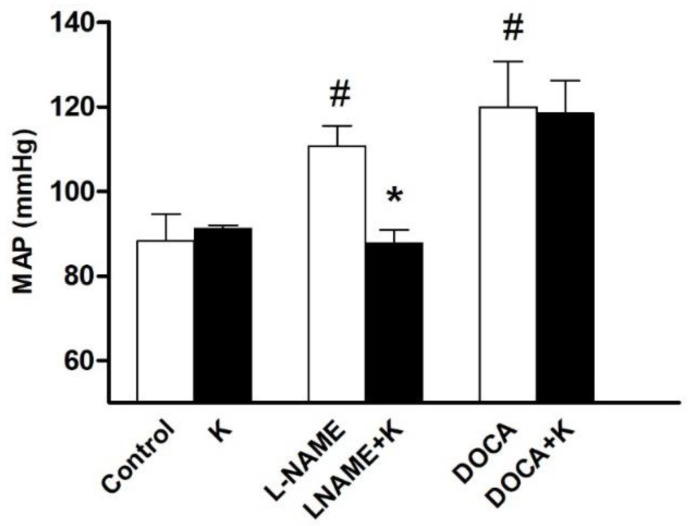
Effect of potassium supplementation on mean arterial blood pressure (MAP) in hypertensive rats. Values are the mean ± SEM, *n* = 5. # *p* < 0.05 versus the control; * *p* < 0.05 versus L-NAME.

Three of the six groups received potassium supplementation. Of them, a reduction in blood pressure (BP) was recorded only in the L-NAME-treated (*p* < 0.05) but not DOCA-treated or vehicle-treated groups (Figure 1). The MAP was 111 ± 5 mmHg in L-NAME versus 88 ± 3 mmHg in the L-NAME+K group (*p* < 0.05) and 88 ± 6 mmHg in the control (Figure 1).

This was a decrease of 20% in the L-NAME group, recovering to a blood pressure like the control group. SBP and DBP were decreased by 15% and 23%, respectively (*p* < 0.05), with no change in pulse pressure in this group (Table 1).

Although a decrease in pulse pressure (PP) was recorded in the DOCA group after potassium supplementation, the blood pressures did not change. The MAP in the DOCA+K group was comparable to the DOCA group (Figure 1).

### 3.2. Potassium Supplementation Reduced the Contractile Response to PE in L-NAME and DOCA-Salt Hypertensive Models

**Figure 2:**
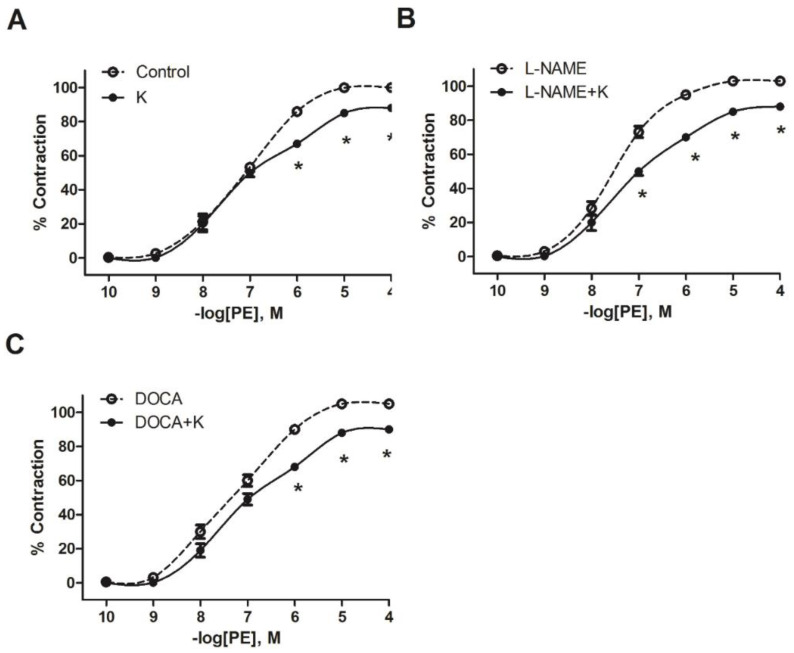
Effect of potassium supplementation on the contractile response of isolated aortic rings to phenylephrine (PE) in normotensive (**A**), L-NAME-induced hypertensive (**B**), and DOCA-induced hypertensive (**C**) rats. Values are the mean ± SEM, *n* = 5. * *p* < 0.05 versus the control, L-NAME, and DOCA.

To test whether the blood pressure effects were related to changes in vascular reactivity, contractile responses to phenylephrine were recorded. The curves for the unsupplemented L-NAME and DOCA hypertensive groups were shifted to the left of the curve for the normotensive control. When the curves for the groups that received potassium supplements were compared with the corresponding groups that were not supplemented, the curves for the supplemented groups were shifted to the right in all cases (Figure 2A–C).

Maximal contraction to PE was significantly decreased after potassium supplementation in all groups: by 16% in vehicle-K, 18% in L-NAME+K, and 17% in DOCA+K (Table 2). The sensitivity (pEC_50_) to PE significantly (*p* < 0.05) decreased in the L-NAME+K group but not in the DOCA+K group (Table 2).

### 3.3. Potassium Supplementation Did Not Improve ACh Relaxation in L-NAME and DOCA-Salt Hypertensive Models

**Figure 3:**
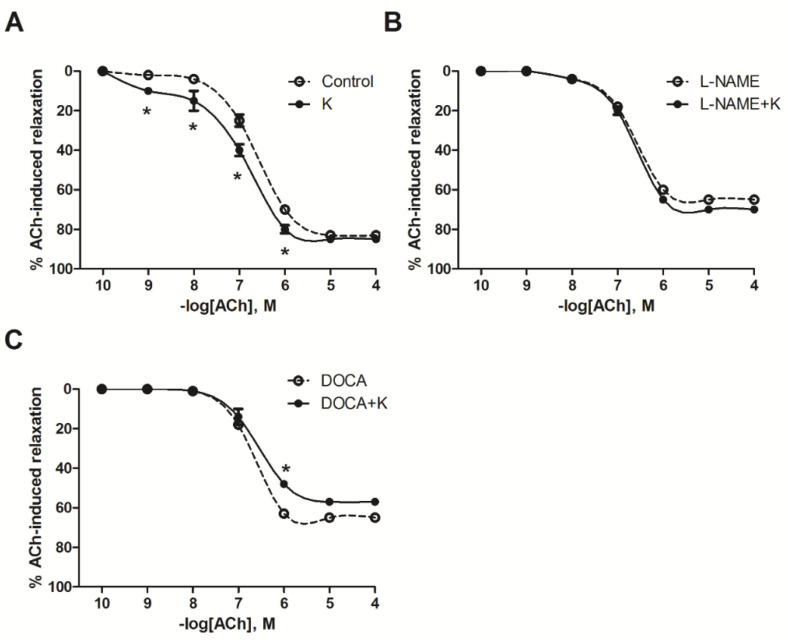
Effect of potassium supplementation on endothelium-dependent relaxation in response to acetylcholine (Ach) in normotensive (**A**), L-NAME-induced hypertensive (**B**), and DOCA-induced hypertensive (**C**) rats. Values are the mean ± SEM, *n* = 5. * *p* < 0.05 versus the control or DOCA.

ACh relaxation was recorded in each group as part of the evaluation of the supplement’s effect on vascular reactivity. First, the hypertensive L-NAME and DOCA models showed a significant decrease in endothelium-dependent vasodilation compared with the control group (60 ± 1% L-NAME, 63 ± 1% DOCA, versus 70 ± 1% for the control; 10^−6^ M ACh; *p* < 0.05) (Figure 3). Second, in both hypertensive groups, L-NAME and DOCA, the vasodilation in response to ACh was not improved by potassium supplementation; it was in fact slightly worsened in the DOCA+K group (65 ± 1% DOCA vs. 57 ± 1% with DOCA+K; 10^−6^ M ACh; *p* < 0.05; Figure 3C).

In contrast, the ACh relaxation curve for the normotensive group that received the potassium supplement was shifted to the left of the un-supplemented control group (70 ± 1% control vs. 80 ± 2% with K, 10^−6^ M ACh; *p* < 0.05, Figure 3A). The sensitivity (pEC_50_) to ACh was significantly (*p* < 0.05) enhanced in the normotensive +K group but not in the NAME+K and DOCA+K groups when compared with respective un-supplemented matched groups (Table 3).

### 3.4. Serum Electrolyte, Creatinine, and Urea Levels

Serum electrolyte, creatinine, and urea levels were measured to determine whether renal function was affected by potassium supplementation that could contribute to its blood pressure effect. Serum was collected after centrifugation of blood at 6000 rpm for 10 min and stored (−2 to −8 °C) for measurement of urea, creatinine, and electrolytes. The potentiometric detection technology (ARCHITECT^®^ c8000 System, Abbott Architect, Abbot Park, IL, USA) method was used in the analysis of the serum at the Chemical Pathology Laboratory, Department of Pathology, University of the West Indies.

**Figure 4:**
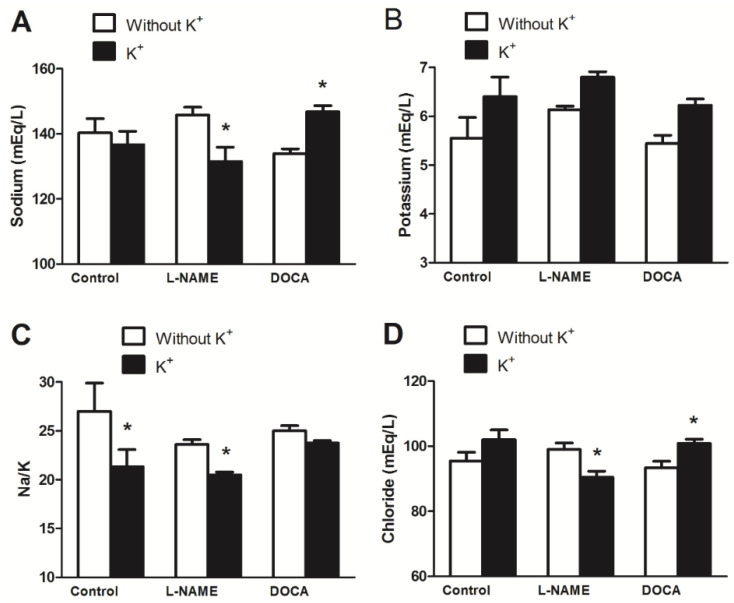
Effect of potassium supplementation on serum electrolyte levels [(**A**) sodium, (**B**) potassium, (**C**) sodium/potassium ratio, and (**D**) chloride] in normotensive and hypertensive rats. Values are the mean ± SEM, *n* = 5. * *p* < 0.05 versus without K^+^.

**Figure 5:**
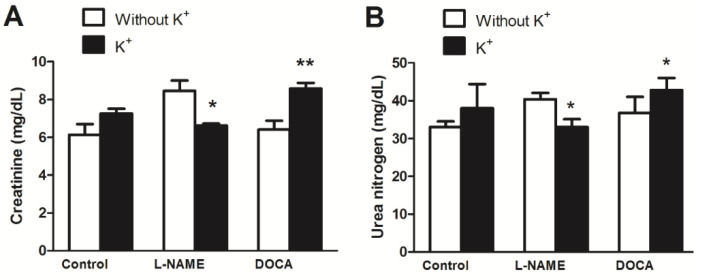
Effect of potassium supplementation on serum creatinine (**A**) and urea nitrogen (**B**) levels in normotensive and hypertensive rats. Values are the mean ± SEM, *n* = 5. * *p* < 0.05 versus without K^+^; ** *p* < 0.01 versus without K^+^.

Sodium, chloride, creatinine, and urea concentrations decreased in the L-NAME+K group but increased in the DOCA+K group when compared with the corresponding unsupplemented groups (Figure 4 and Figure 5). Serum sodium was 146 ± 2 mg/dL in the L-NAME group vs. 132 ± 4 mg/dL in the L-NAME+K group (*p* < 0.05; Figure 4A), the Na/K ratio was 24 ± 0.5 in the L-NAME group vs. 21 ± 0.3 in the L-NAME+K group (*p* < 0.05; Figure 4C), and serum chloride was 99 ± 2 mg/dL in the L-NAME group vs. 91 ± 2 mg/dL in the L-NAME+K group (*p* < 0.05; Figure 4D).

The serum creatinine level was 8.5 ± 0.5 mg/dL in the L-NAME group vs. 6.6 ± 0.1 mg/dL in the L-NAME+K group (*p* < 0.05, Figure 5A) while the level of urea nitrogen was 40 ± 2 mg/dL in the L-NAME group vs. 33 ± 2 mg/dL in the L-NAME+K group (*p* < 0.05, Figure 5B). However, these decreases were not found in the DOCA group or increased in the cases of sodium, chloride, creatinine, and urea.

### 3.5. Serum Malondialdehyde Level

**Figure 6:**
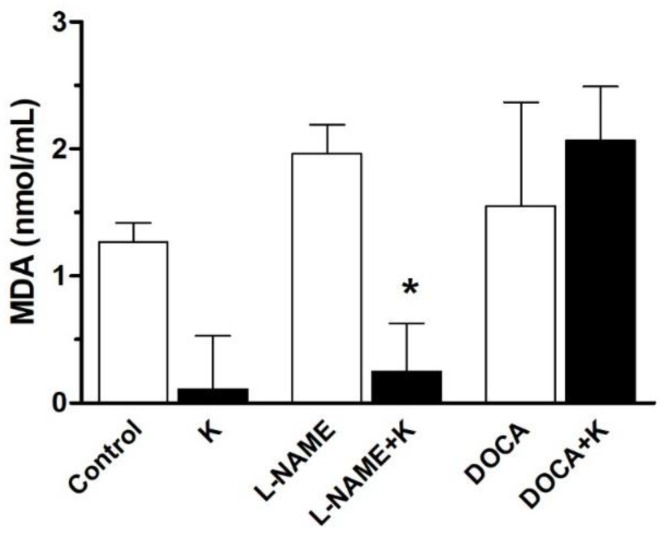
Serum concentration of lipid peroxidation product, MDA (nmol/mL), of the experimental groups, obtained after 8 weeks of treatment. Values are the mean ± SEM, *n* = 5. * *p* < 0.05 versus L-NAME.

Thiobarbituric acid reactive substances (TBARS) are an index of lipid peroxidation and oxidative stress levels in various tissues, a feature associated with hypertension [11,20,23]. The OXItek TBARS Assay kit was used to assess lipid peroxidation levels. A fluorometer was used to detect fluorescent analysis at excitation of 530–550 nm, from which a standard fluorometer curve was plotted, and MDA concentrations for each specimen were determined. The concentrations of the serum lipid peroxidation product, malondialdehyde, for the groups are shown in Figure 6, and show a significant (*p* < 0.05) decrease in the L-NAME+K group but not in the DOCA+K group.

### 3.6. Body Weight and Glomerular Filtration Rate

During the 8-week treatment period, the body weights of the rats were monitored weekly. During this time, all rats exhibited increased body weight. At the end of the 8-week period, rats in the hypertensive groups showed a smaller increase in body weight than the control group (Table 4).

The highest eGFR (estimated glomerular filtration rate (a key index of kidney function; 4545.03) represents a healthy baseline for the control and best kidney function. L-NAME is known to induce hypertension by inhibiting nitric oxide, which may affect renal perfusion. Our observations were lower weight and the lowest eGFR (2693.13) and higher creatinine and urea levels, suggesting worsened kidney function (Table 5). Potassium supplementation for this group showed a slight improvement in weight and eGFR compared to L-NAME alone, which suggests a partial protective effect of potassium. DOCA is typically used to induce salt-sensitive hypertension. There was a slight drop in eGFR (4177.49), with a mild kidney impact, which may not be as damaging as L-NAME in this case. With potassium supplementation (DOCA + K⁺ Group), eGFR is further reduced (3072.38). Adding K⁺ did not improve and may have worsened renal function slightly. K⁺ treatment alone or combined with L-NAME or DOCA shows variable, sometimes modest, protective or neutral effects. There was a statistically significant difference in eGFR values between the groups (*p* < 0.001). This strongly suggests that the treatment groups differ in their effects on kidney function.

## 4. Discussion

There are inconsistencies in reports on the efficacy of potassium supplementation in the management of hypertension [24]. This study demonstrates that K supplementation is differentially effective in lowering blood pressure in hypertension. Potassium depletion has been shown to result in substantial impairment of cardiac function in dogs and healthy human subjects. Hypokalemia can alter cardiac structure, contributing to cardiac necrosis and fibrosis in both experimental animals and humans. Potassium supplementation, on the other hand, appears to offer protective effects for both the heart and kidneys. Increased potassium intake and the prevention of hypokalemia have been linked to reduced blood pressure in hypertensive individuals and animal models. Other studies have shown that correction of hypokalemia through dietary potassium adaptation reduces DOCA/salt-induced cardiac hypertrophy and other forms of dysfunction [25,26].

Its efficacy appears to be dependent on the model and phase of hypertension. The data showed that 8 weeks of supplementation caused reduced blood pressure in the L-NAME hypertensive rats but not in the DOCA-salt hypertensive rats. The L-NAME and DOCA-uninephrectomy (DOCA-UNX) models not only differ in how they induce hypertension but also in their molecular signaling pathways. In our study, potassium was associated with reduced vasoconstrictory response [27]. However, this effect was observed in both hypertensive models and the normotensive control, suggesting that hypo-reactivity alone could not explain the differences in the effect of potassium supplementation on blood pressure. Though more prominent with the L-NAME model, perhaps because this model inhibits NOS (especially eNOS), it is also associated with increased renin and angiotensin II levels and contributes to vasoconstriction, hypertrophy, and oxidative stress, while the DOCA-UNX model shows indirect NO reduction via endothelial damage, suppressed RAAS (low renin model), and elevated angiotensin levels (from DOCA), acting independently of renin, and drives inflammation, fibrosis, and sodium reabsorption [10,11]. Moreover, ACh relaxation was unaltered in both models of hypertension, which rules out action via enhanced endothelium-dependent vasorelaxation. Alterations in NO-independent relaxation pathways or receptor desensitization, which could be occasioned by increased oxidative stress, may blunt vasodilation and downgrade endothelial signaling pathways. Potassium supplementation may fail if target channels become dysfunctional.

Potassium caused a more significant reduction in oxidative stress and ROS signaling through a decrease in TBARS [28], which can reduce blood pressure [29]. This was more prominent in the L-NAME model, perhaps because this model inhibits NO and promotes superoxide (O_2_⁻) accumulation, a moderate inflammatory response, and antioxidant enzymes (e.g., SOD) but increases NADPH oxidase activity, while the DOCA-UNX model shows a more robust inflammatory and fibrotic response when compared with the L-NAME model, directly increasing NADPH oxidase expression (via mineralocorticoid receptor signaling) and mitochondrial ROS levels and boosting ROS production, leading to vascular and renal injury [10,11,28].

Potassium supplementation is reported to result in a reduced Na^+^/K^+^ ratio and mean arterial blood pressure [30], and it decreased the serum Na^+^/K^+^ ratio in the L-NAME model because there were no major changes in renal sodium transporters; as such, sodium retention is not a primary feature. But not so in the DOCA-UNX model, wherein ENaC (epithelial sodium channel) expression was upregulated in the distal nephron, which enhances Na⁺ reabsorption, induces blood volume expansion, and affects Na⁺/K⁺-ATPase activity. There is substantial evidence that potassium depletion, or hypokalemia, in clinical and experimental findings, has a negative impact on the cardiovascular system as well as the kidney and contributes to the pathogenesis of hypertension and renal injury [25,26]. This is consistent with reported improvement in eGFR and BP for the L-NAME-treated group along with K+ salt supplementation but not for DOCA-salt plus K+ supplementation. The lack of improvement in this group could be due to unforeseen negative interactions between DOCA and the K+ supplement [25].

Potassium supplementation had a more prominent effect on pulse pressure, which is associated with cardiac remodeling [26,31] and the downregulation of TGF-β1, collagen genes, and other fibrotic markers in the DOCA-UNX, perhaps due to the fibrotic and inflammatory effects of mineralocorticoid excess, when compared with the L-NAME model. In DOCA-salt hypertensive mice, however, potassium supplementation did not decrease blood pressure, although it prevented cardiac hypertrophy [26,32].

Our results suggest that potassium supplementation can lower blood pressure, improve endothelial function, reduce oxidative stress, and enhance kidney function through a reduction in creatinine levels and glomerular filtration rates (GFRs) [5] through the modulation of sodium handling as high K⁺ intake downregulates sodium reabsorption in the nephron (especially via the Na⁺-Cl⁻ cotransporter), reducing volume overload and pressure on glomeruli. Our estimated GFR showed a decrease in the DOCA-UNX group but not in the L-NAME group. This could be because in the early phase, the GFR may initially increase or remain stable due to compensatory hyperfiltration by the remaining kidney, with glomerular damage from high pressure, interstitial fibrosis, and tubular atrophy occurring in the chronic phase. As this model mimics progressive kidney disease and is often used to study chronic kidney disease (CKD) development in the setting of salt-sensitive hypertension, it is possible that our 8-week exposure may not have captured this chronic pathogenesis. The differences seen with the models are due to the underlying pathogenesis. The L-NAME model decreases both the initial and chronic GFR outcomes, but the DOCA-UNX model decreases the chronic phases of the GFR, with variable outcomes of hyperfiltration in the initial phases [33,34].

Although changes in heart rate (HR) do not appear to contribute to hypertension in these models [22], supplementation had an effect on pulse pressure in the DOCA group, which suggests that the effect on cardiac contractility cannot be ruled out [31]. Moreover, potassium supplementation prevented cardiac hypertrophy in DOCA-salt hypertensive mice [26]. However, there does not appear to be a cardiac component to the blood pressure-lowering effect of supplementation in the L-NAME model. A reduction in eGFR, with increases in creatinine and urea levels, validates the disease model (e.g., the DOCA-salt model mimicking hypertension-related kidney damage) or reduced renal blood flow (e.g., L-NAME blocking nitric oxide, lowering perfusion) and typically signifies worsening kidney function.

Higher dietary potassium levels are linked to lower BP and reduced cardiovascular risk in human RCTs, blunted BP elevation, reduced renal damage in animal models, and lower CKD progression risk in epidemiological presentations, and adequate potassium has been recommended for blood pressure and kidney health, unless contraindicated. This is because it can reduce markers of kidney inflammation and fibrosis, counteract sodium’s effect, and reduce vascular stiffness through the enhancement of endothelial function [10,11]. In models like L-NAME-induced hypertension, in which nitric oxide is suppressed, potassium may help offset reduced vasodilation. Potassium can enhance renal tubular function, improving the kidney’s ability to excrete waste like urea and creatinine, which may help maintain a higher eGFR or slow its decline. It may lower levels of aldosterone, a hormone linked to sodium retention, renal fibrosis, and hypertension. Our results also suggest that potassium has antioxidant and anti-inflammatory effects [35], so it reduces oxidative stress in the kidneys. This protects nephrons (filtering units) from damage [17].

Using molecular markers such as eNOS, mineralocorticoid receptor (MR) signaling, SOD, and NADPH oxidase is a powerful way to mechanistically support observations of impaired vasorelaxation in experimental hypertension, especially in the context of dietary potassium interventions. These markers help to quantify, localize, and mechanistically explain the involvement of endothelial, oxidative, and inflammatory pathways. The absence of renal histology, providing information about glomerulosclerosis index and fibrosis scoring, limits the interpretation of kidney protection. Finally, although the sample size for each group was calculated statistically, the number of animals used for each group suggests cautious interpretation of the results. There are limitations to this study, and we hope to address them in subsequent studies.

## 5. Conclusions

In conclusion, the antihypertensive effect of potassium supplementation is dependent on the type and phase of hypertension [14,17]. It is more effective in L-NAME-like models but not in DOCA-salt-like models of hypertension and involves more than a direct vascular effect to include improved renal function and reduced oxidative stress, possibly because of the pathogenesis presentations during the acute and chronic phases of hypertension development.

The sub-phenotyping of hypertension may suggest that dietary potassium may be more effective in patients with endothelial dysfunction-dominant hypertensive subtypes compared with volume-dependent hypertension (e.g., low-renin and salt-sensitive) and may call for K⁺ supplementation studies stratified by hypertension subtype as a refining clinical guideline.

## Figures and Tables

**Table 1:** Effect of potassium supplementation on systolic blood pressure (SBP), diastolic blood pressure (DBP), and pulse pressure (PP) of normotensive and experimental hypertensive rats with DOCA or L-NAME.

Parameter	Control	K	L-NAME	L-NAME+K	DOCA	DOCA+K
**SBP, mmHg/s**	114 ± 7	116 ± 3	137 ± 5 ^#^	116 ± 2 *	135 ± 10 ^##^	140 ± 7
**DBP, mmHg/s**	76 ± 6	78 ± 1	98 ± 5 ^#^	75 ± 4 *	96 ± 11 ^#^	104 ± 9
**PP, mmHg**	39 ± 1	39 ± 3	40 ± 1	41 ± 1	48 ± 1 ^###^	36 ± 1 ***

Values are the mean ± SEM of 5 experiments in mmHg. ^#^ *p* < 0.05, ^##^ *p* < 0.01, and ^###^ *p* < 0.001 versus the control; * *p* < 0.05 versus L-NAME, *** *p* < 0.001 versus DOCA.

**Table 2:** Effect of potassium supplementation on contractile response to PE (10^−10^ to 10^−4^ M) in the intact aortic rings of normotensive rats and hypertensive rats with DOCA or L-NAME.

	Control	K^+^	L-NAME	L-NAME+K	DOCA	DOCA+K
**Emax. (%)**	98 ± 3	82 ± 5 *	101 ± 2	83 ± 4 *	102 ± 5	85 ± 5 *
**pEC_50_ (M)**	7.07 ± 0.11	7.20 ± 0.19	7.48 ± 0.07	7.19 ± 0.16 *	7.19 ± 0.16	7.12 ± 0.18

The values represent the maximal effect (Emax. %) and half maximal effective concentration (pEC_50_ M) to PE. The values represent the mean of 5 independent experiments, and are the mean ± standard error of the mean (SEM). Statistically significant difference * *p* < 0.05 vs. the control, L-NAME, or DOCA.

**Table 3:** Effect of potassium supplementation on relaxation induced by ACh (10^−10^ to 10^−4^ M) in the intact aortic rings of normotensive rats and hypertensive rats with DOCA or L-NAME.

	Control	K^+^	L-NAME	L-NAME+K	DOCA	DOCA+K
**Emax. (%)**	84 ± 1	86 ± 3	67 ± 2	72 ± 2	68 ± 3	58 ± 1
**pEC50 (M)**	6.64 ± 0.03	6.91 ± 0.10 *	6.67 ± 0.09	6.69 ± 0.09	6.70 ± 0.13	6.58 ± 0.05

The values represent the maximal effect (Emax. %) and half maximal effective concentration (pEC50 M) to PE. The values are the mean of 5 independent experiments and represent mean ± standard error of the mean (SEM). Statistically significant difference * *p* < 0.05 vs. the control.

**Table 4:** Body weight for each experimental group for the 8-week duration of treatment.

Parameter	Control	K	L-NAME	L-NAME+K	DOCA	DOCA+K
**Mean initial weight (g)**	281 ± 27	250 ± 30	210 ± 15	240 ± 18	279 ± 33	230 ± 74
**Mean final weight (g)**	409 ± 32	377 ± 13	304 ± 13	324 ± 21	381 ± 23	357 ± 39
**Mean weight change (g)**	128 ± 59	127 ± 43	94 ± 28	84 ± 39	102 ± 56	127 ± 113
**% change**		**(−)1%**		**(−)11%**		**(+)25%**

Values are the mean ± SEM of 5 experiments.

**Table 5:** eGFR calculations for each experimental group for the 8-week duration of treatment.

Groups		Weight	W^0.695^	Creatinine	C^−0.660^	Urea	U^−0.391^	eGFR
**Control**	880	409	65.34	6	0.31	32	0.26	4545.03
**K^+^**	880	377	61.74	7	0.28	37	0.24	3665.29
**L-NAME**	880	304	53.16	8.5	0.24	40	0.24	2693.13
**L-NAME + K^+^**	880	324	55.57	6.2	0.30	32	0.26	3782.85
**DOCA**	880	381	62.19	6	0.31	35	0.25	4177.49
**DOCA + K^+^**	880	357	59.44	8.5	0.24	38	0.24	3072.38

eGFR = 880 × W^0.695^ × C^−0.660^ × U^−0.391^ (eGFR is estimated GFR (µL/min), W is weight (g), U is urea (mmol/L), and C is creatinine concentration (µmol/L).

## Data Availability

The original contributions presented in this study are included in the article. Further inquiries can be directed to the corresponding authors.

## Abbreviations

ACh	Acetylcholine
ANOVA	Analysis of variance
BP	Blood pressure
CKD	Chronic kidney disease
DBP	Diastolic blood pressure
DOCA	Deoxycorticosterone acetate
eGFR	Estimated glomerular filtration rate
GFR	Glomerular filtration rates
HR	Heart rate
L-NAME	L-NG-Nitro-arginine methyl ester
MAP	Mean arterial blood pressure
MDA	Malondialdehyde
NOS	Nitric oxide synthase
PP	Pulse pressure
RBF	Reduced renal blood flow
SBP	Systolic blood pressure
SEM	Standard error of the mean
UNX	Uninephrectomy
